# Supplementary figures and images for: In silico analysis of the transcriptional regulatory logic of neuronal identity specification throughout the C. elegans nervous system
Source: eLife. 2021 Jun 24;10:e64906. doi: 10.7554/eLife.64906 (PMC8225391; doi:10.7554/eLife.64906)

Figure 2- Figure Supplement 1

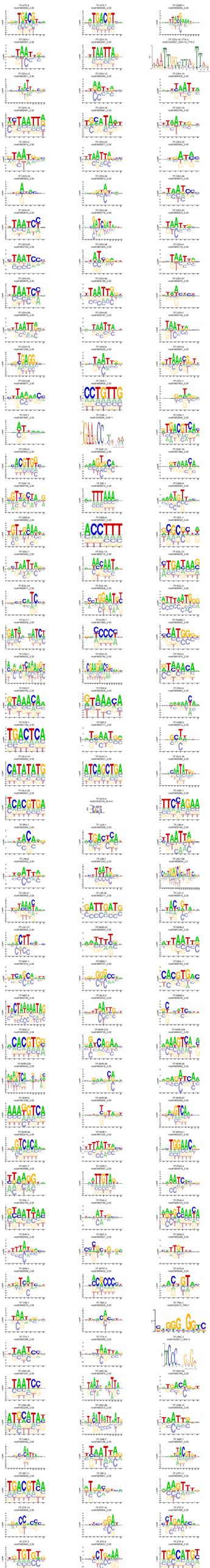

Supplement: Figure 2—source data 1. — Images were downloaded from cisbp.ccbr.utoronto.ca/. See motif source information in Supplementary file 1A. [file elife-64906-fig2-data1.pdf]
