# Supplementary material for: In silico analysis of the transcriptional regulatory logic of neuronal identity specification throughout the C. elegans nervous system
Source: eLife. 2021 Jun 24;10:e64906. doi: 10.7554/eLife.64906 (PMC8225391; doi:10.7554/eLife.64906)
Supplement: Figure 7—source data 1. [file elife-64906-fig7-data1.pdf]

ADA

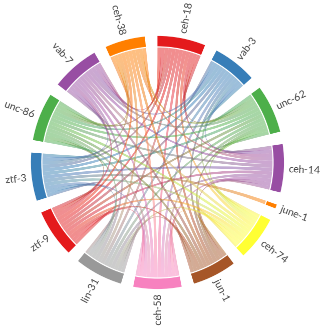

made with chord pro

ADE

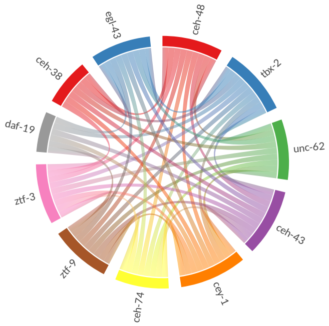

made with chord pro

ADF

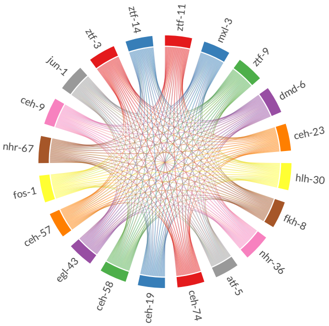

made with chord pro

ADL

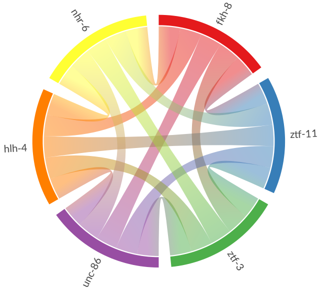

made with chord pro

AFD

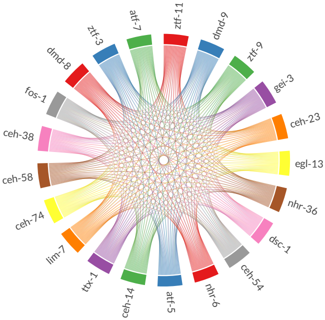

made with chord pro

AIA

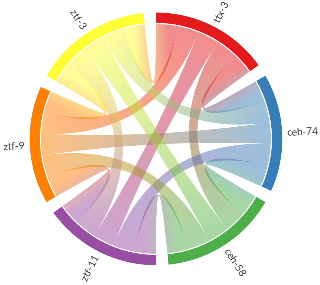

made with chord pro

AIB

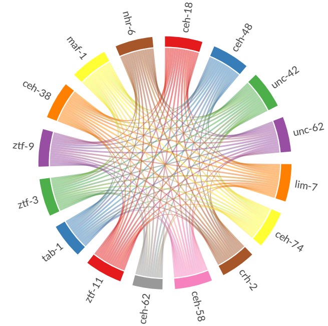

made with chord pro

AIM

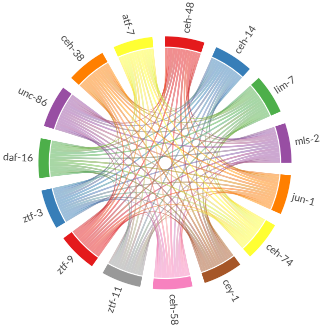

made with chord pro

AIN

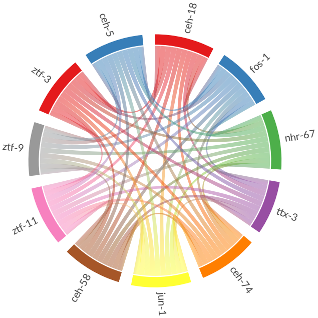

made with chord pro

AIY

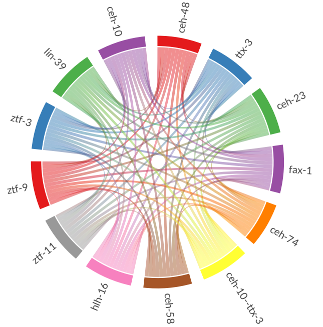

made with chord pro

AIZ

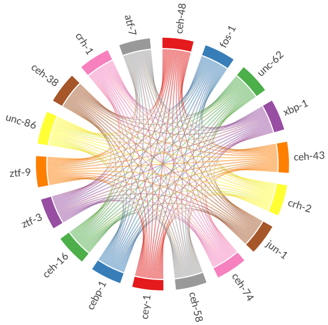

made with chord pro

ALA

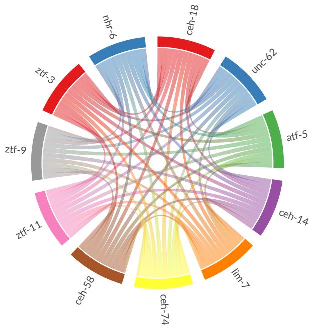

made with chord pro

ALM

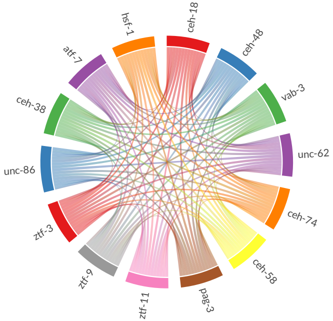

made with chord pro

ALN

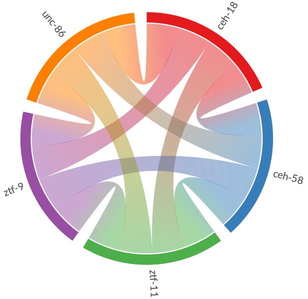

made with chord pro

AQR

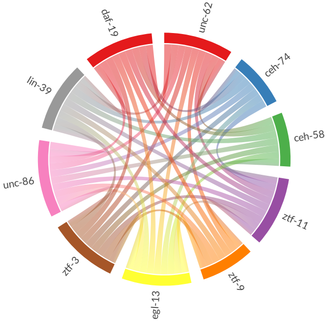

made with chord pro

ASE

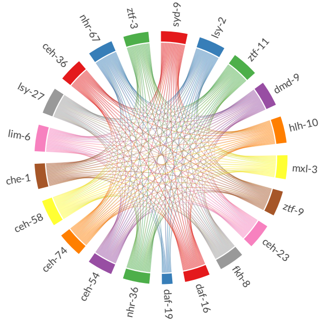

made with chord pro

ASG

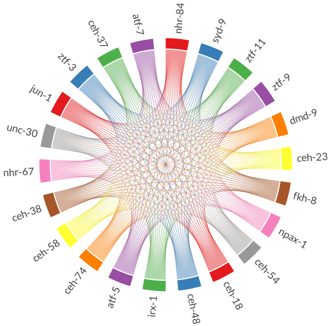

made with chord pro

ASH

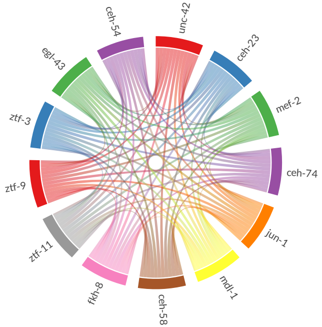

made with chord pro

ASI

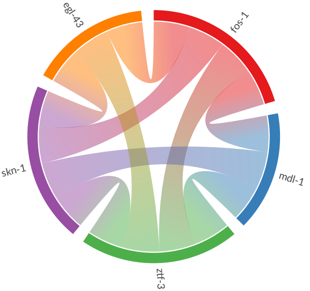

made with chord pro

ASJ

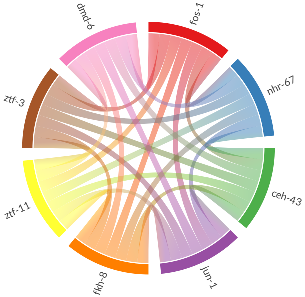

made with chord pro

ASK

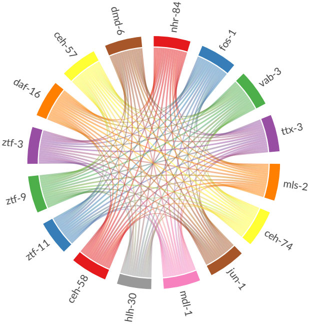

made with chord pro

AS

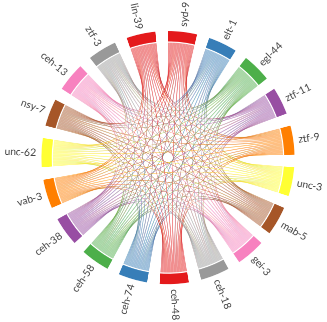

made with chord pro

AUA

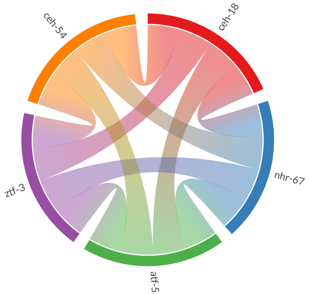

made with chord pro

AVA

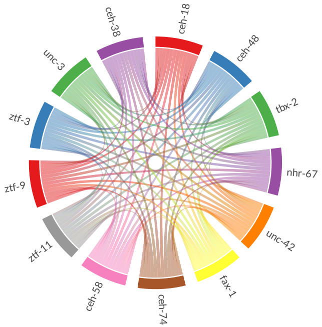

made with chord pro

AVB

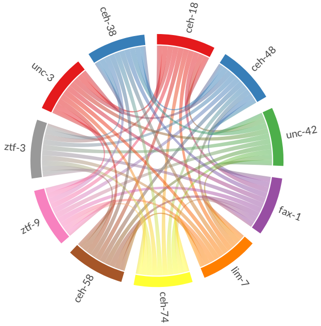

made with chord pro

AVD

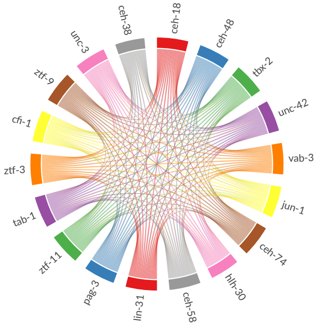

made with chord pro

AVE

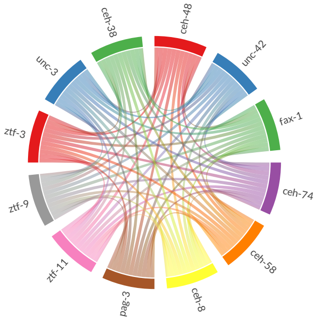

made with chord pro

AVF

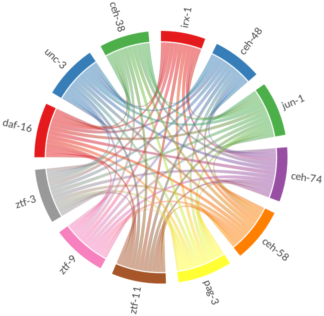

made with [chord pro]

AVG

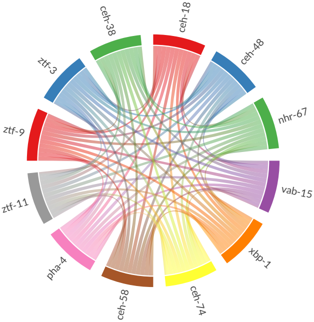

made with [chord pro]

AVH

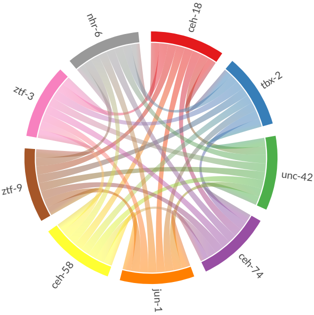

made with [chord pro]

AVJ

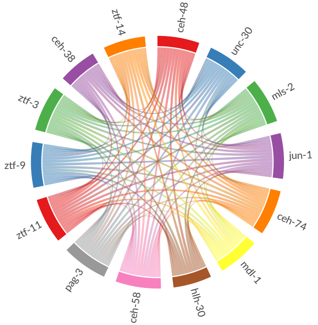

made with chord pro

AVK

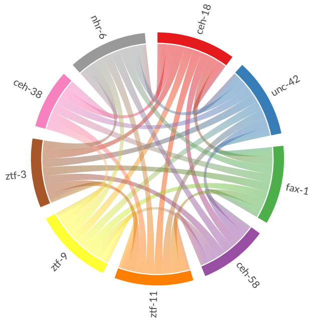

made with chord pro

AVL

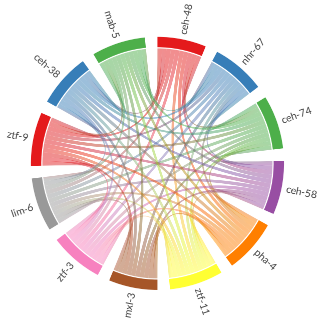

made with chord pro

AVM

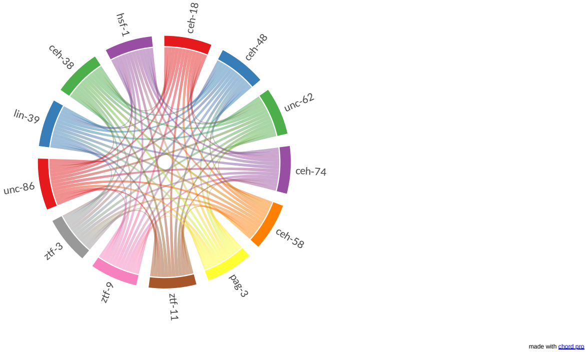
ceh-18
ceh-48
unc-62
ceh-74
ceh-58
pag-3
ztf-11
ztf-9
ztf-3
unc-86
lin-39
ceh-38
hsf-1
made with chord pro

AWA

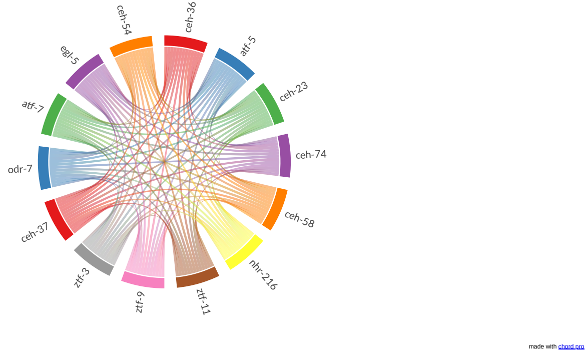
ceh-36
atf-5
ceh-23
ceh-74
ceh-58
nhr-216
ztf-11
ztf-9
ztf-3
ceh-37
odr-7
atf-7
egl-5
ceh-54
made with chord pro

AWB

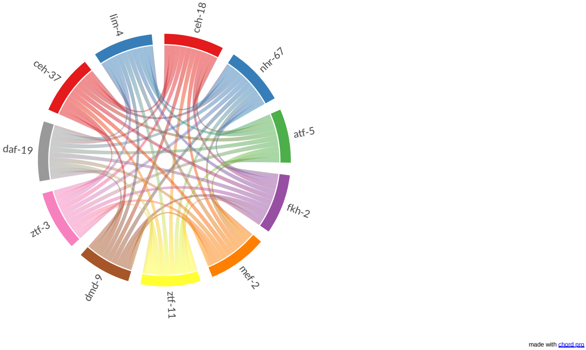
ceh-18
nhr-67
atf-5
fkh-2
mef-2
ztf-11
dmd-9
ztf-3
daf-19
ceh-37
lim-4
made with chord pro

AWC

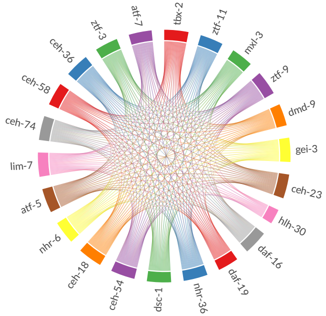

made with chord pro

BAG

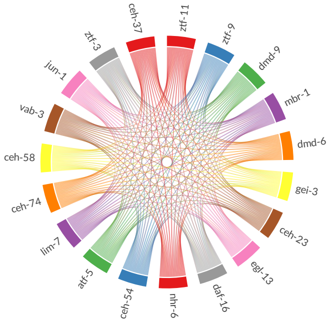

made with chord pro

BDU

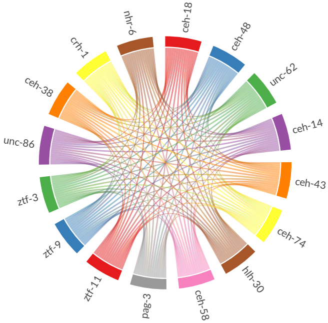

made with chord pro

CAN

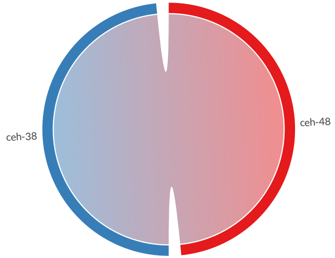

made with chord pro

CEP

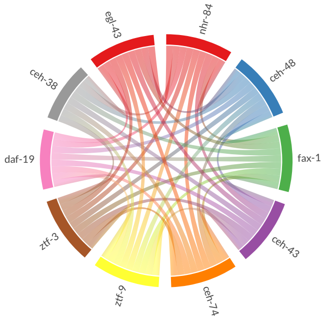

made with chord pro

DA

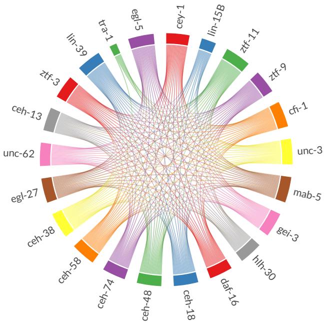

made with chord pro

DB

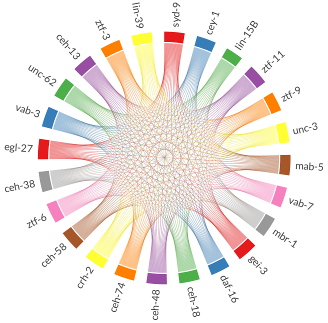

made with chord pro

DVA

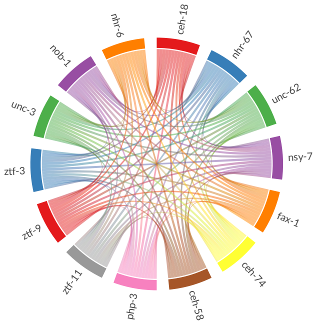

made with chord pro

DVB

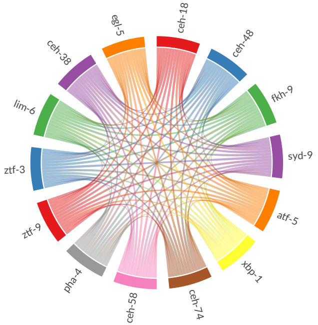

made with chord pro

DVC

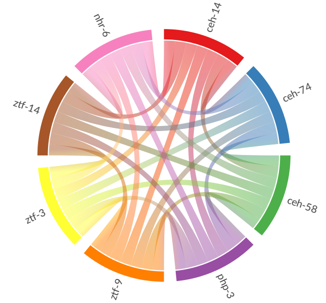

made with chord pro

FLP

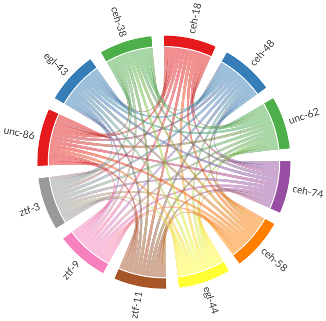

made with chord pro

HSN

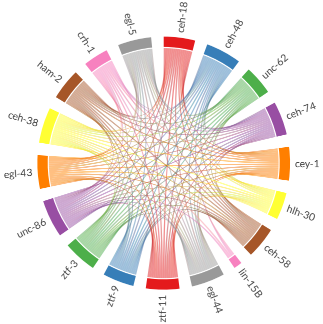

made with chord pro

I1

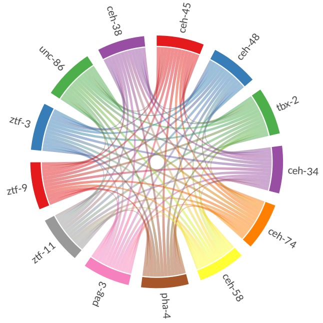

made with chord pro

I2

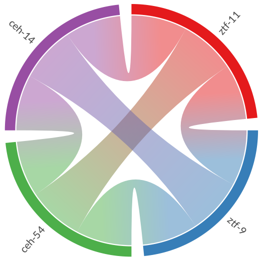

made with chord pro

I3

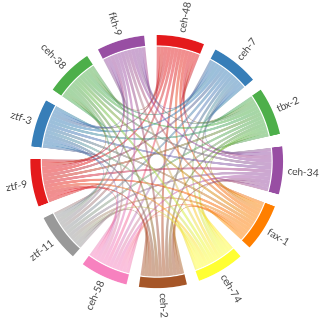

made with chord pro

I4

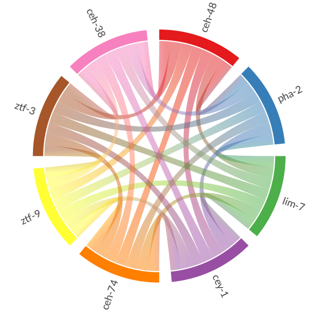

made with chord pro

I5

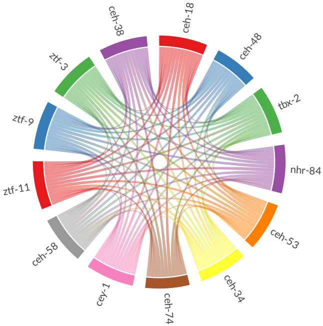

made with chord pro

IL2

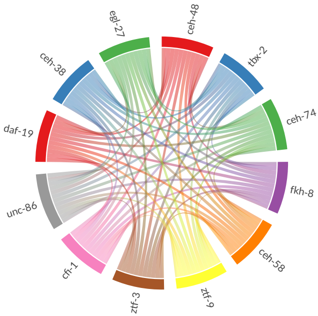

made with chord pro

LUA

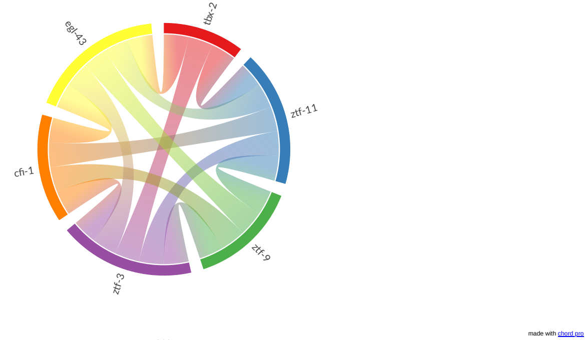
tbx-2
ztf-11
ztf-9
ztf-3
cfi-1
egl-43
made with chord pro

M1

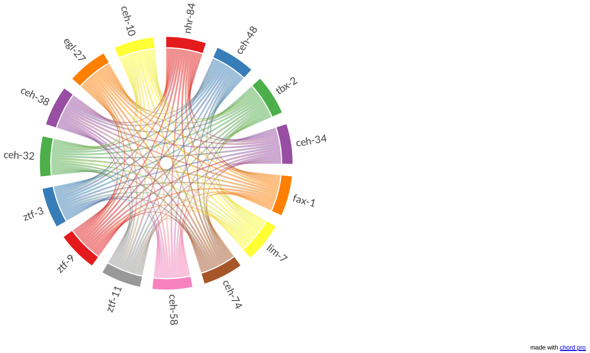
nhr-84
ceh-48
tbx-2
ceh-34
fax-1
lim-7
ceh-74
ceh-58
ztf-11
ztf-9
ztf-3
ceh-32
ceh-38
egl-27
ceh-10
made with chord pro

M2

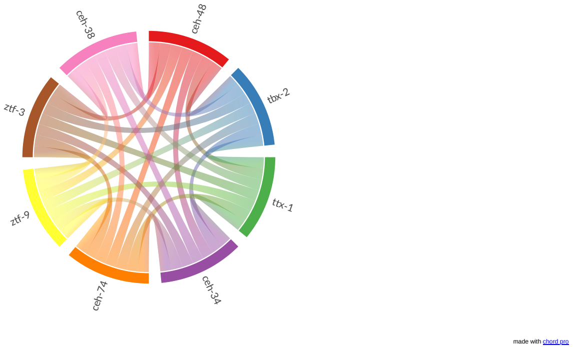
ceh-48
tbx-2
ttx-1
ceh-34
ceh-74
ztf-9
ztf-3
ceh-38
made with chord pro

M3

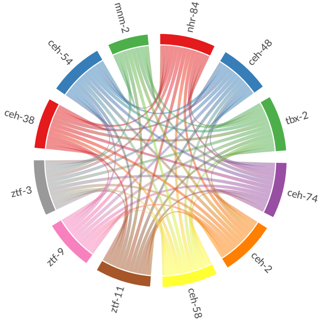

made with chord pro

M4

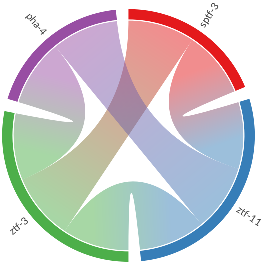

made with chord pro

M5

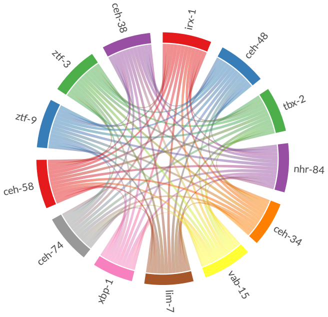

made with chord pro

MC

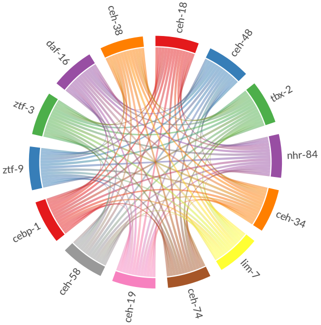

made with chord pro

MI

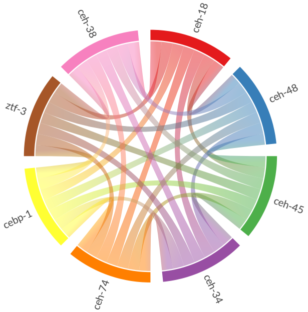

made with chord pro

NSM

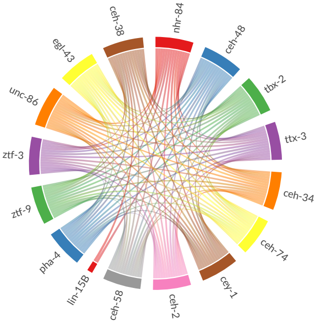

made with chord pro

OLL

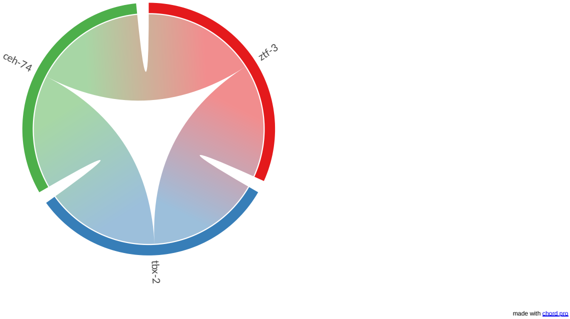
ceh-74
ztf-3
tbx-2
made with chord pro

PDA

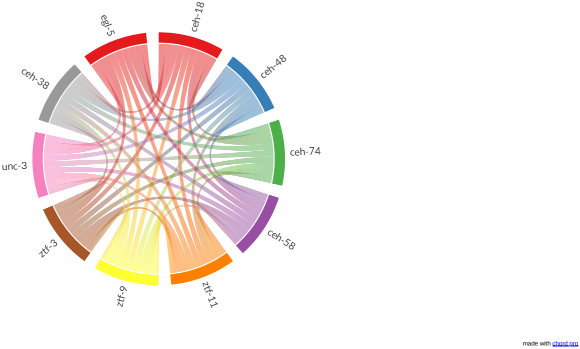
egl-5
ceh-18
ceh-48
ceh-38
ceh-74
unc-3
ceh-58
ztf-3
ztf-9
ztf-11
made with chord pro

PDB

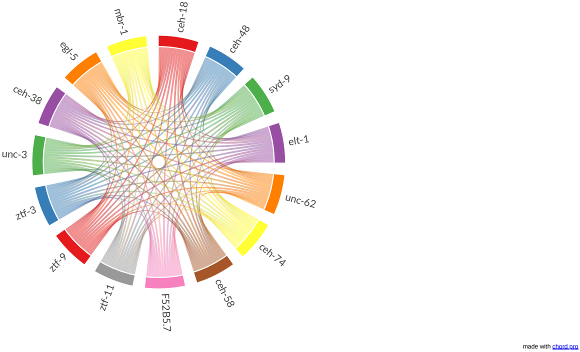
mbr-1
ceh-18
ceh-48
egl-5
syd-9
ceh-38
elt-1
unc-3
unc-62
ztf-3
ceh-74
ztf-9
ztf-11
F52B5.7
ceh-58
made with chord pro

PDE

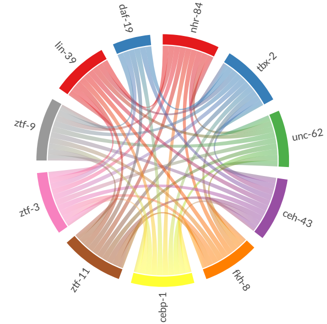

made with chord pro

PHA

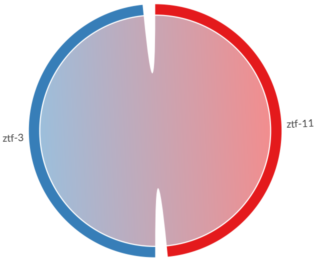

made with chord pro

PHC

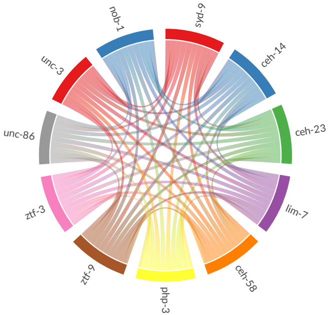

made with chord pro

PLM

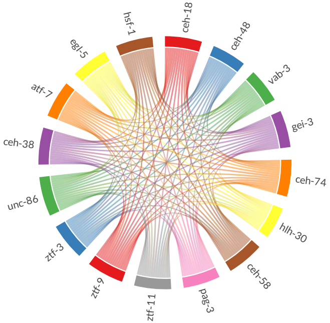

made with chord pro

PLN

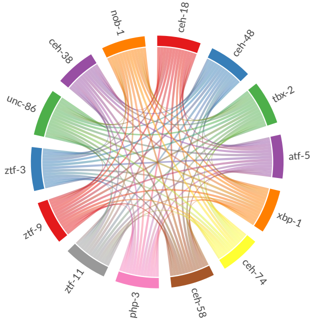

made with chord pro

PQR

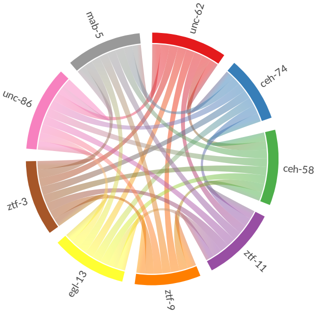

made with chord pro

PVC

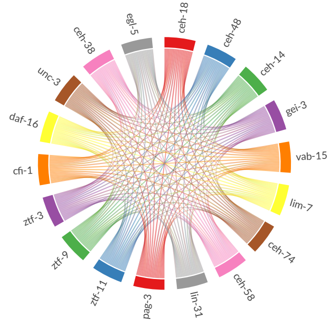

made with chord pro

PVD

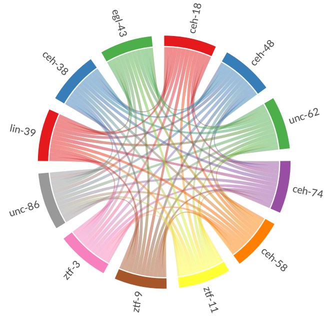

made with chord pro

PVM

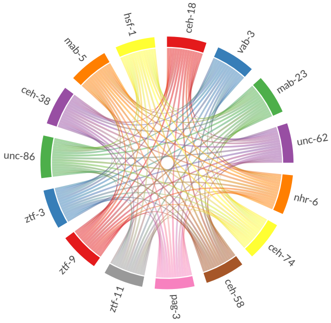

made with chord pro

PVN

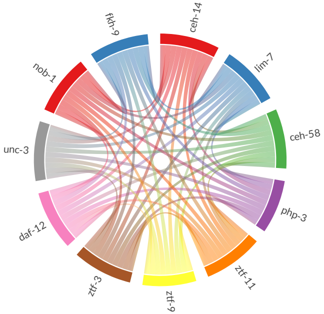

made with chord pro

PVP

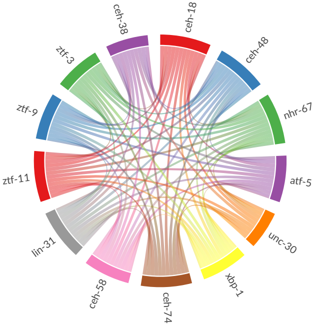

made with chord pro

PVQ

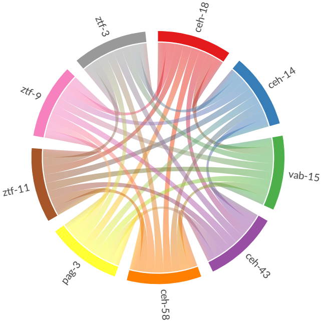

made with chord pro

PVR

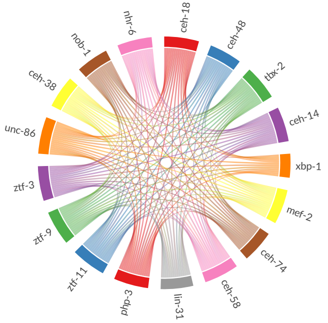

made with chord pro

PVT

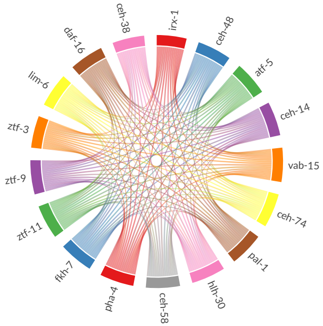

made with chord pro

PVW

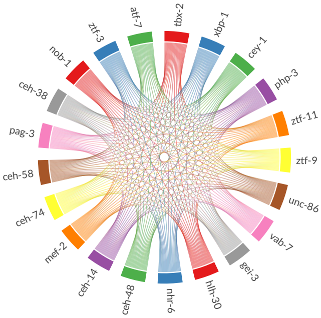

made with chord pro

RIA

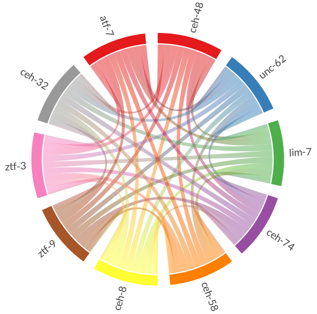

made with chord pro

RIB

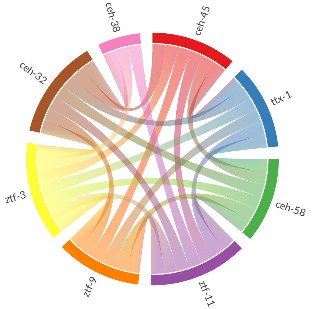

made with chord pro

RIC

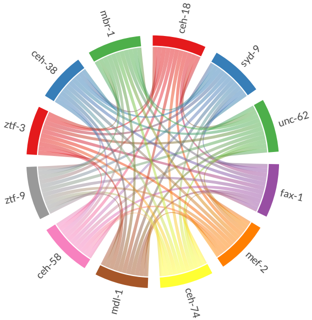

made with chord pro

RID

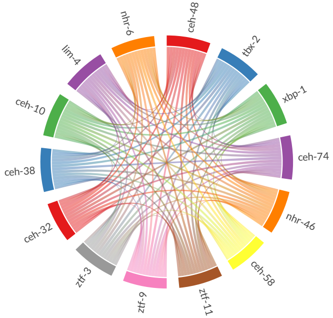

made with chord pro

RIF

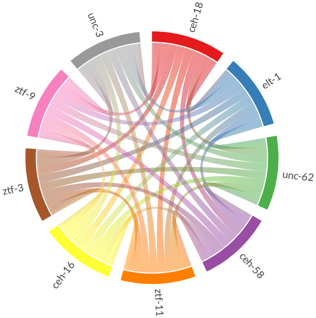

made with chord pro

RIG

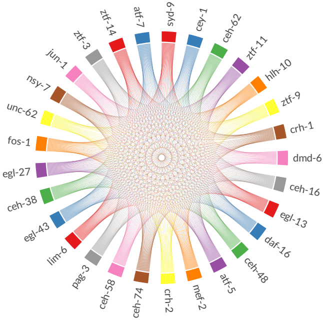

made with chord pro

RIH

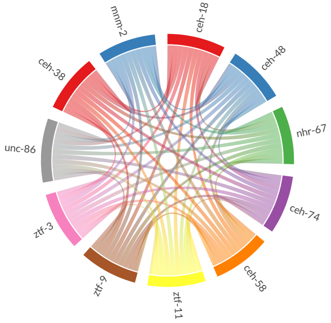

made with chord pro

RIM

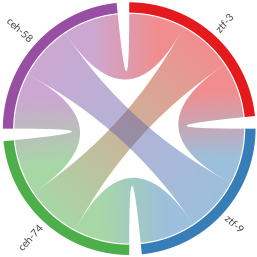

made with chord pro

RIP

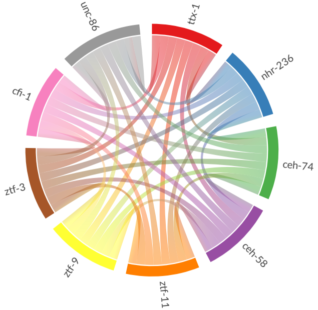

made with chord pro

RIR

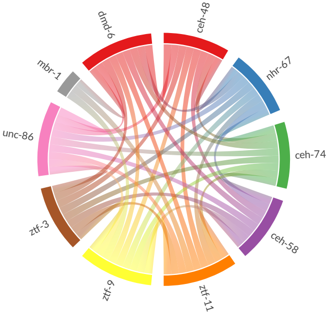

made with chord pro

RIS

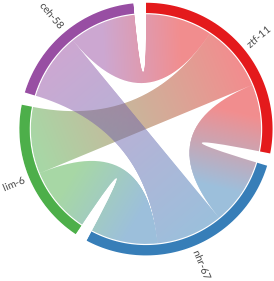

made with chord pro

RIV

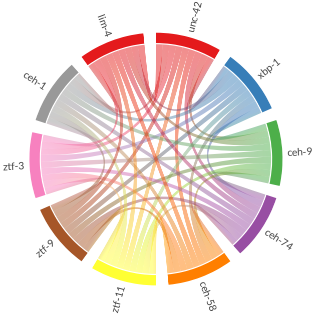

made with chord pro

RMD

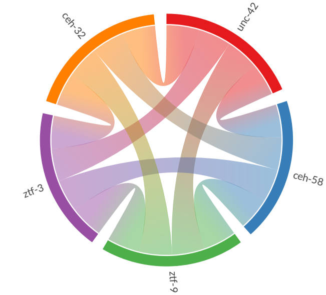

made with chord pro

RME

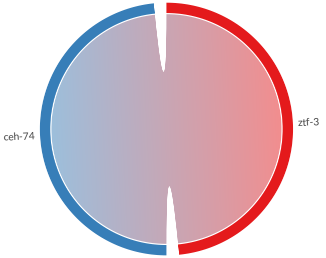

made with chord pro

RMF

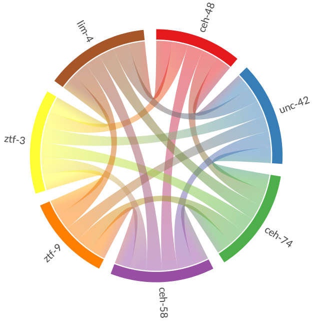

made with chord pro

RMG

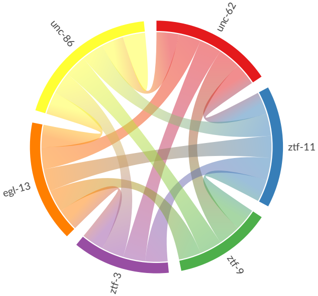

made with chord pro

RMH

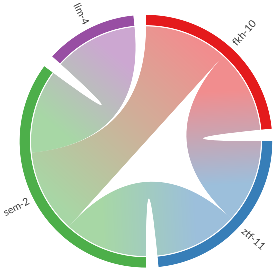

made with chord pro

SAA

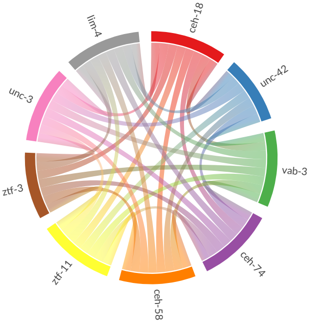

made with chord pro

SAB

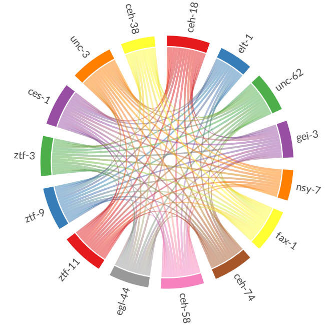

made with chord pro

SDQ

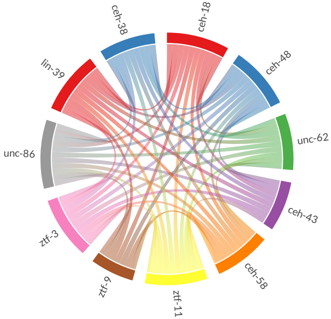

made with chord pro

SIA

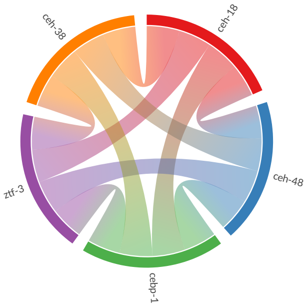

made with chord pro

SIB

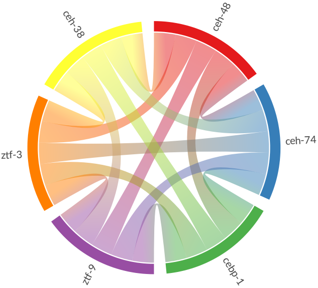

made with chord pro

SMB

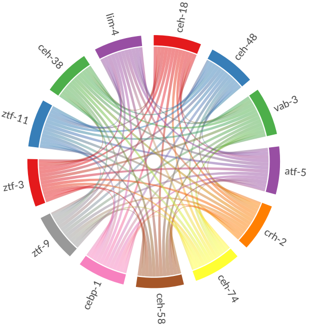

made with chord pro

SMD

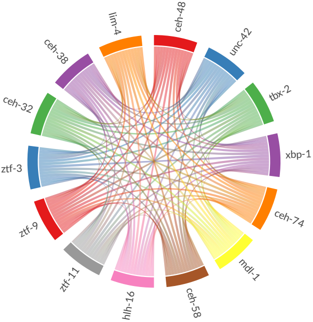

made with chord pro

URA

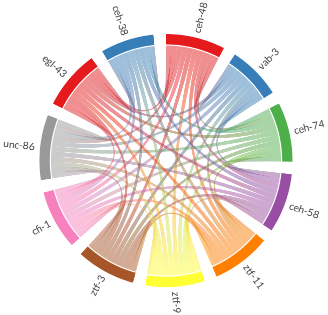

made with chord pro

URB

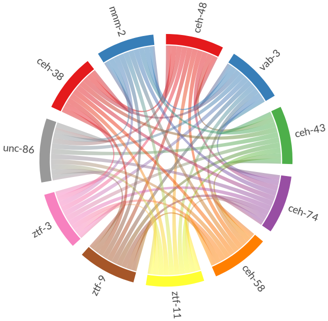

made with chord pro

URX

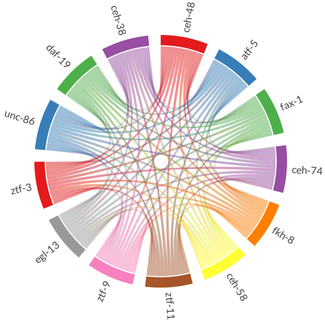

made with chord pro

URY

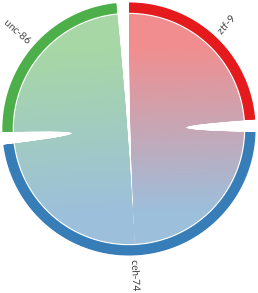

made with chord pro

VA

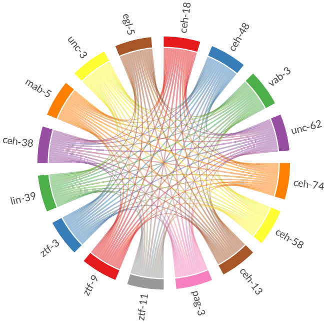

made with chord pro

VB

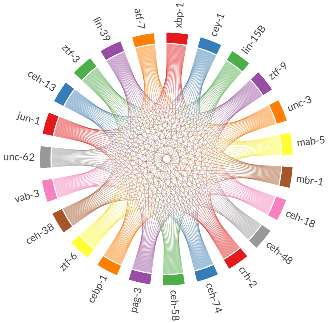

made with chord pro

VC

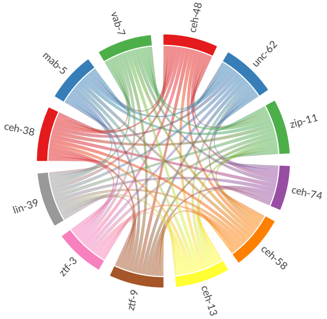

made with chord pro

VD_DD

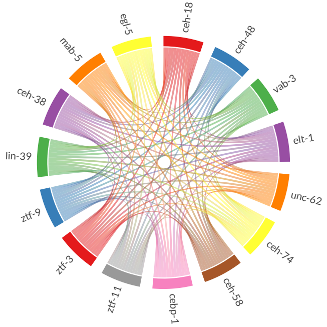

made with chord pro
